# Proteolytic biomarkers are related to prognosis in COPD- report from a population-based cohort

**DOI:** 10.1186/s12931-018-0772-5

**Published:** 2018-04-12

**Authors:** Robert Linder, Eva Rönmark, Jamshid Pourazar, Annelie F. Behndig, Anders Blomberg, Anne Lindberg

**Affiliations:** 1 Department of Public Health and Clinical Medicine, Division of Medicine, Umeå University, SE-90187 Umeå, Sweden; 2 Department of Public Health and Clinical Medicine, the OLIN unit, Division of Occupational and Environmental Medicine, Umeå University, SE-90187 Umeå, Sweden

**Keywords:** Chronic obstructive pulmonary disease (COPD), Matrix metalloproteinases, Tissue inhibitor of metalloproteinases, Mortality, Spirometry

## Abstract

**Background:**

The imbalance between proteases and anti-proteases is considered to contribute to the development of COPD. Our aim was to evaluate the protease MMP-9, the antiprotease TIMP-1 and the MMP-9/TIMP-1-ratio as biomarkers in relation to prognosis. Prognosis was assessed as lung function decline and mortality. This was done among subjects with COPD in a population-based cohort.

**Methods:**

In 2005, clinical examinations including spirometry and peripheral blood sampling, were made in a longitudinal population-based cohort. In total, 1542 individuals participated, whereof 594 with COPD. In 2010, 1031 subjects participated in clinical examinations, and 952 subjects underwent spirometry in both 2005 and 2010. Serum MMP-9 and TIMP-1 concentrations were measured with enzyme linked immunosorbent assay (ELISA). Mortality data were collected from the Swedish national mortality register from the date of examination in 2005 until 31st December 2010.

**Results:**

The correlation between biomarkers and lung function decline was similar in non-COPD and COPD, but only significant for MMP-9 and MMP-9/TIMP-1-ratio in non-COPD. Mortality was higher in COPD than non-COPD (16% vs. 10%, *p* = 0.008). MMP-9 concentrations and MMP-9/TIMP-1 ratios in 2005 were higher among those who died during follow up, as well as among those alive but not participating in 2010, when compared to those participating in the 2010-examination. In non-COPD, male sex, age, burden of smoking, heart disease and MMP-9/TIMP-1 ratio were associated with increased risk for death, while increased TIMP-1 was protective. Among those with COPD, age, current smoking, increased MMP-9 and MMP-9/TIMP-1 ratio were associated with an increased risk for death.

**Conclusions:**

The expected association between these biomarkers and lung function decline in COPD was not confirmed in this population-based study, probably due to a healthy survivor effect. Still, it is suggested that increased proteolytic imbalance may be of greater prognostic importance in COPD than in non-COPD.

**Electronic supplementary material:**

The online version of this article (10.1186/s12931-018-0772-5) contains supplementary material, which is available to authorized users.

## Background

Chronic obstructive pulmonary disease, COPD, is today considered a heterogeneous syndrome, including several clinical phenotypes [1]. Under-diagnosis is common; around 20–30% of all cases are identified by healthcare. Most of the current knowledge regarding the pathophysiological mechanisms in COPD is based on highly selected study populations [2, 3]. Thus, it is unclear to what extent the results are generalisable to COPD in the population.

The discovery of alpha-1-antitrypsin deficiency (AATD) and its association with emphysema in smokers provided the concept of imbalance between proteases and anti-proteases and its contribution to COPD development [4]. Cross-sectional studies on selected COPD-populations have observed increased levels of matrix metalloproteinase-9 (MMP-9) in COPD, and an association between the level of MMP-9 and FEV_1_ [5–7]. We have recently shown an inverse relationship between FEV_1_ and serum MMP-9 in a cross-sectional study of a population-based COPD-cohort [8]. This indicates that increased proteolytic activity is associated with disease severity not only in selected COPD populations. However, MMP-9 should be considered in relation to the tissue inhibitor of metalloproteinases-1 (TIMP-1), because of its inhibiting effect on MMP-9 [6, 9].

There are a few longitudinal studies within this topic, of small and selected study populations. These studies indicate that MMP-9 is related to lung function decline in COPD [10]. Also, in a selected population of patients with AATD-associated emphysema, higher plasma MMP-9 levels were associated with disease progression [11]. Still, the link between proteolytic imbalance and long-term COPD disease progress has not yet been evaluated in population-based studies. We believe that our population-based COPD cohort provides an excellent basis for a longitudinal follow-up, when it comes to addressing the prognostic value of MMP-9 in COPD [8].

The first aim of this population-based study was to evaluate the biomarkers MMP-9, TIMP-1 and MMP-9/TIMP-1 ratio in serum. The second aim was to put this in relation to disease progress and prognosis, assessed as lung function decline and mortality respectively. The third aim was to do this among subjects with and without COPD.

## Methods

### Participants and design

The Obstructive Lung Disease in Northern Sweden (OLIN) COPD study includes all 993 subjects with COPD (defined as FEV_1_/VC < 0.7) together with 993 age and sex-matched controls without obstructive lung function impairment identified during re-examination of population-based cohorts in 2002–04. The study population (*n* = 1986) has since 2005 been annually invited to a basic examination program. The current study population includes all 1542 subjects with complete data on structured interview, spirometry and blood sampling participating in the 2005-examinations. Study design, recruitment of study population, participation and basic characteristics in 2005, are previously described in detail [8, 12].

**Fig. 1:**
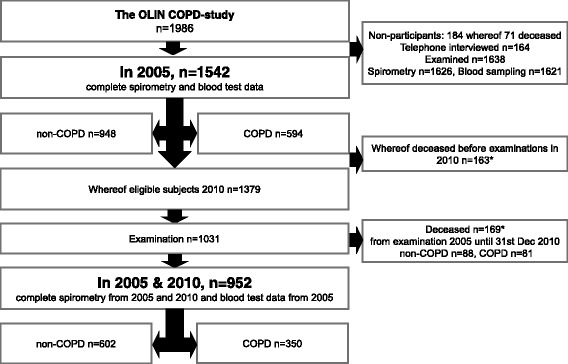
Flowchart of the study population

Follow-up spirometry data were collected from the 2010-examinations of the cohort under study. All-cause mortality data were collected from the Swedish national mortality register from the date of examination in 2005 until end of December 2010. In 2010, 1379 out of the 1542 were still alive, and 1031 of them participated in clinical examination. In total 952 subjects participated in the clinical examinations, including spirometry, in both 2005 and 2010 (Fig. 1). The Regional Ethics Committee at Umeå University approved the study, which was carried out according to the declaration of Helsinki. All participants provided written informed consent before the performance of any study-related assessments.

### Structured interview questionnaire

Previously validated questions regarding respiratory symptoms were used in a structured interview [13–15]. In addition, data on smoking habits and co-morbidities, such as heart disease (defined as one or more of either; angina pectoris, myocardial infarction, coronary artery bypass graft (CABG) surgery, percutaneous coronary intervention (PCI) and chronic heart failure), were included.

### Spirometry; spirometric classification and definitions of decline in lung function

A Mijnhardt Vicatest 5 dry spirometer was used to obtain lung function data, following the ATS guidelines of procedure [16]. A reversibility test was carried out if FEV_1_/highest of FVC or SVC < 0.70, or if FEV_1_ < 80% of predicted value. COPD was defined as FEV_1_ divided by FVC or SVC < 0.70, using the highest values pre- or post-bronchodilation. Disease severity was classified according to the GOLD (Global Initiative for Obstructive Lung Disease) spirometric criteria based on FEV_1_% predicted; GOLD 1–4 [17]. Local reference values were used for FEV_1_ [18].

Lung function change from 2005 until 2010, assessed as change of FEV_1_ using the highest value of FEV_1_ either pre- or post-bronchodilation, was calculated as:

1. The mean FEV_1_ decline (ml/year); the unadjusted difference in FEV_1_ in ml (absolute value of FEV_1_ 2010 - absolute value of FEV_1_ 2005 divided by number of years of observation time, based on person days).
2. Annual change in units of percent predicted normal value of FEV_1_. (FEV_1_% predicted 2010 - FEV_1_% predicted 2005 divided by number of years of observation time, based on person days) [19].

### Laboratory analysis

The handling and analyses of blood samples have been described previously [8]. Samples were stored since 2005 at − 20 **°**C, thereafter thawed and analysed simultaneously in 2013. Serum concentrations of MMP-9 and TIMP-1 were assayed using commercially available ELISA kits (DuoSet® ELISA Development System, R&D Systems Europe Ltd., United Kingdom), according to the manufacturer’s instructions.

### Statistical analysis

Dichotomous variables were analysed using Pearson’s χ2 and continuous variables with the Mann–Whitney U-test since they did not meet the criterion of normal distribution. Non-normally distributed data are presented as median and interquartile range (IQR). Mortality risk is expressed as hazard ratio (HR) and analysed in a Cox-regression model. The IBM SPSS Statistics for Macintosh, version 23 (IBM Corp., Armonk, N.Y., USA) was used for all analyses and *P*-values < 0.05 were considered significant.

## Results

### Basic characteristics and biomarker serum concentrations among participants in 2005

The study population from 2005 included in total 1542 subjects, whereof 594 with COPD (Table 1). The distribution by GOLD grade among those with COPD was 64% GOLD 1, 32% GOLD 2 and 4% GOLD 3–4. The serum concentration of MMP-9 (median, interquartile range, (IQR)) was significantly lower in non-COPD than COPD; 505 (364–606) vs. 535 (315–653) ng/ml, *p* = 0.017. Contrastingly, the concentration of TIMP-1 and the MMP-9/TIMP-1-ratio were similar in non-COPD and COPD; 316 (220–490) vs. 304 (227–430) ng/ml, *p* = 0.252 and 1.36 (0.85–2.09) vs. 1.50 (0.83–2.32), *p* = 0.168. Basic characteristics are previously published in the referred publication from the same study [8].

**Table 1:** Study population, basic characteristics in 2005, comparing non-COPD and COPD

	Non-COPD *n* = 948	COPD *n* = 594	P
Female, n (%)	447 (47)	248 (42)	**0.038**
Age (years), median (IQR)	67 (55–71)	69 (57–71)	**0.015**
BMI (kg/m^2^), median (IQR)	27 (24–30)	26 (24–29)	**< 0.001**
Non-smoker, n (%)	443 (47)	149 (25)	**< 0.001**
Ex-smoker, n (%)	384 (41)	247 (42)	0.676
Current smoker, n (%)	120 (13)	197 (33)	**< 0.001**
Pack years, median (IQR)	0.8 (0–12)	14 (0–27)	**< 0.001**
FEV_1_% predicted, median (IQR)	1.03 (0.93–1.13)	0.85 (0.73–0.96)	**< 0.001**
Productive cough, n (%)	212 (22)	238 (40)	**< 0.001**
Heart disease, n (%)	149 (16)	111 (19)	1.000

*IQR* Inter quartile range. Significant *p*-values in bold

### Annual lung function decline

The cohort generated 5174 person-years and was followed for a median (IQR) of 5.53 (5.21–5.64) years. In total, 952 subjects, whereof 424 (45%) women, participated in the clinical examinations including spirometry in both 2005 and 2010. The median (IQR) annual decline in FEV_1_ was similar in COPD and non-COPD, − 40 (− 64– − 22) and − 46 (− 68– − 16) ml/year respectively, (*p* = 0.428). The annual decline in percent-units of FEV_1_ percent predicted was less in non-COPD than COPD; − 0.21 (− 1.10–0.51) vs. -0.54 (− 1.43–0.50), *p* = 0.020.

### Relation between biomarker values and lung function decline

The correlation between biomarker levels and annual lung function decline, when using change in ml FEV_1_ as well as change in units of FEV_1_ percent predicted value, had a similar pattern in non-COPD and COPD. Though it was significant for MMP-9 and MMP-9/TIMP-1-ratio only in non-COPD (Table 2).

**Table 2:** Linear regression analysis of biomarker levels 2005 in relation to lung function decline, in non-COPD and COPD respectively

Change in:	non-COPD		COPD	
ml FEV_1_	Beta	*P*	Beta	*P*
MMP-9	**−0.098**	**0.016**	−0.063	0.240
TIMP-1	**0.100**	**0.014**	0.025	0.648
MMP-9/TIMP-1 ratio	**−0.128**	**0.002**	−0.072	0.183
units of FEV_1_ percent predicted value				
MMP-9	**−0.117**	**0.004**	−0.091	0.089
TIMP-1	0.056	0.169	0.009	0.864
MMP-9/TIMP-1-ratio	**−0.109**	**0.007**	−0.086	0.110

Significant values in bold

A non-response analysis was carried out, comparing biomarker values in 2005 between subjects participating and not participating in examination with spirometry in 2010 (deceased were excluded from the analyses) (Fig. 1). The analysis showed a higher level of MMP-9 and an increased MMP-9/TIMP-1 ratio along with a decreased TIMP-1 concentration in 2005 when comparing non-participants and participants at examination in 2010. This was evident in both non-COPD and COPD (Additional file 1: Table S1).

### Biomarkers, mortality and risk factors for death

In total, 169 individuals died during the observation time. The cumulative mortality was lower in non-COPD, 10% (*n* = 88), than in COPD, 16% (*n* = 81), *p* = 0.008. In both non-COPD and COPD subjects, serum MMP-9 as well as MMP-9/TIMP-1 ratio in 2005 were higher among those who died during the observation time compared to among those who were alive and eligible for follow-up in 2010 (Table 3). Both MMP-9 level and MMP-9/TIMP-1 ratio were higher among deceased subjects with COPD than among deceased non-COPD individuals (Table 4).

**Table 3:** Comparing serum biomarker levels 2005 among survivors and subjects deceased during the observation time, in non-COPD and COPD respectively

Biomarkers	non–COPD		P	COPD		P
	Participating *n* = 860	Deceased *n* = 88		Participating *n* = 513	Deceased *n* = 81	
	Median (IQR)	Median (IQR)		Median (IQR)	Median (IQR)	
MMP-9 (ng/ml)	501 (346–605)	535 (428–628)	**0.026**	514 (210–641)	607 (493–681)	**< 0.001**
TIMP-1 (ng/ml)	311 (224–521)	327 (250–381)	0.670	300 (222–479)	311 (260–366)	0.839
MMP-9/TIMP-1 ratio	1.34 (0.81–2.09)	1.71 (1.19–2.16)	**0.001**	1.38 (0.76–2.29)	1.86 (1.52–2.34)	**< 0.001**

*IQR* Inter quartile range. Significant *p*-values in bold

**Table 4:** Comparing serum biomarker levels 2005 between deceased in non-COPD and COPD during the observation time

Biomarkers	non–COPD Deceased *n* = 88	COPD Deceased *n* = 81	*P*
	Median (IQR)	Median (IQR)	
MMP–9 (ng/ml)	534.5 (427.5–628)	607 (493–681)	**0.001**
TIMP–1 (ng/ml)	327 (250–381)	311 (260–366)	0.488
MMP–9/TIMP-1-ratio	1.71 (1.19–2.16)	1.86 (1.52–2.34)	**0.020**

*IQR* Inter quartile range. Significant *p*-values in bold

Univariate analyses revealed that increasing age, male sex and heart disease as well as increasing levels of MMP-9 and MMP-9/TIMP-1 ratio were associated with an increased risk for death, expressed as Hazard Ratio (HR, 95% CI). This was the case in both non-COPD and COPD, while smoking status was a significant risk factor for death only among non-COPD subjects. Productive cough and lower FEV_1_ were each associated with a higher risk for death in COPD (Additional file 1: Table S2).

In a multivariate model, male sex, age, ex- and current smoking, heart disease, decreased TIMP-1 and increased MMP-9/TIMP-1 ratio were significantly associated with increased risk for death in non-COPD subjects, while age, current smoking as well as increased MMP-9 and MMP-9/TIMP-1 ratio were associated with increased risk for death among those with COPD (Table 5). These results remained significant also when adjusted for lung function assessed as FEV_1_% predicted (Additional file 1: Table S3) and productive cough respectively (Additional file 1: Table S4). When smoking status was replaced by pack-years, MMP-9/TIMP-1 ratio was no longer significant in non-COPD but remained significant in COPD and so did MMP-9 (Additional file 1: Table S5).

**Table 5:** Risk for death expressed as HR (95% CI), analyses stratified for non-COPD and COPD in a Cox regression model adjusting for sex, age, smoking status and heart disease

	Non-COPD *n* = 948	COPD *n* = 594
	HR (95% CI)	HR (95% CI)
Female	1	1
Male	**1.700** (1.067–2.708)	1.538 (0.938–2.523)
Age^a^	**1.105** (1.078–1.134)	**1.112** (1.083–1.142)
Non-smoker	1	1
Ex-smoker	**2.182** (1.318–3.611)	1.296 (0.709–2.370)
Current smoker	**4.917** (2.357–10.257)	**2.429** (1.296–4.552)
No heart disease	1	1
Heart disease	**2.443** (1.574–3.792)	1.361 (0.843–2.197)
MMP-9^a,b^	1.001 (1.000–1.003)	**1.003** (1.002–1.004)
TIMP-1^a,b^	**0.997** (0.996–0.999)	0.998 (0.997–1.000)
MMP-9/TIMP-1-ratio^a,b^	**1.099** (1.014–1.190)	**1.299** (1.086–1.555)

Significant values in bold

^a^continuous variable. ^b^added one by one to the multivariate model

## Discussion

In this population-based study, subjects with and without COPD showed a different risk factor pattern when evaluating biomarkers in relation to prognosis, assessed as mortality. In non-COPD, male sex, burden of smoking, heart disease and MMP-9/TIMP-1 ratio increased the risk for death, and serum concentration of TIMP-1 had a protective effect. Meanwhile in COPD, age, current smoking and increased serum concentration of MMP-9 and MMP-9/TIMP-1 ratio increased the risk for death. These findings bring additional verification of MMP-9’s impact on COPD, previously explored in epidemiological, [11, 20–23] as well as recent genetic studies [24] This supports earlier findings of MMP-9’s relation to mortality in other groups of subjects [25, 26]. Though the present results imply that protease-anti-protease imbalance has a prognostic impact both among subjects with and without COPD, their risk factor patterns did differ. In COPD, the biomarker pattern indicates that an increased proteolytic activity is associated with an increased risk for death and the significance of these biomarkers’ importance for the prognosis in COPD merits further evaluation.

Based on the results in a selected population of COPD-patients [10], we expected to find a correlation between the selected biomarkers and lung function decline in COPD, but this was not the case. Still, when comparing the 2005-biomarkers among those who did and those who did not attend in 2010, a greater protease-anti-protease imbalance was indicated among non-participants. Likewise, those who died during follow-up had higher MMP-9 and MMP-9/TIMP-1 ratio in 2005 than those who lived and participated in follow-up, and the levels were higher in COPD than non-COPD. Notwithstanding the association between biomarkers and lung function decline in non-COPD, this could not be proven in COPD. However, we assume that there is an influence also among those with COPD that cannot be demonstrated here, due to the loss of follow up of those with the highest serum biomarker levels. The findings imply a healthy survivor effect, which could offer an explanation to the somewhat unexpected absence of a correlation between biomarkers and lung function decline in COPD, when assessed over a five-year period. The lower mortality and lower biomarker levels associated with mortality in non-COPD than COPD provides a different basis for evaluating decline in lung function in relation to the biomarkers and may contribute to the observed significant relationship in non-COPD.

To the best of our knowledge, this is the first time these biomarkers have been evaluated in relation to prognosis and assessed as risk factors for death in a population-based COPD study. In both groups, MMP-9 levels and MMP-9/TIMP-1 ratio in 2005 were higher among those who died compared to those who survived. Furthermore, among those with COPD, these biomarkers were associated with an increased risk for death, also after adjustment for confounders, and independent of disease severity, assessed as FEV_1_% of predicted. This suggests that markers of increased proteolytic imbalance may be an important disease mechanism in COPD and an issue that deserves further evaluation. In non-COPD subjects, other risk factors appear more consequential as risk factors for death, and TIMP-1 had a protective effect.

Lung function decline in COPD is proposed for evaluation across several years, as FEV_1_ values may naturally fluctuate between different examinations carried out at shorter time intervals [27]. Similar to the last point, the progression of COPD has been found to be heterogeneous and thus a long interval of follow up may be more indicative of lung function decline in a population than a shorter interval [28]. In the current study, decline in lung function was assessed over a period of approximately five years, which can be considered satisfactory.

Phenotyping studies have explained the heterogeneity in COPD and might represent an opportunity to enhance diagnosis, predict outcomes and personalize treatments in patients. Suggested COPD phenotypes are based on clinical parameters such as; emphysema severity measured by CT, spirometry, nutritional status, exercise capacity and exacerbation frequency [29, 30]. Presumably, one parameter could be associated to many phenotypes, and there is probably an overlap between phenotypes in the population. Based on the knowledge of AATD and COPD [4], it seems appropriate to associate markers of increased proteolytic imbalance to greater parenchymal degradation and emphysema development. It is unclear to what extent various parameters and phenotypes define the complex syndrome of COPD, as presented in the population. However, proteolytic imbalance appears to be a factor that is significant on a population level [22, 31], and the results generate hypotheses for future studies of COPD phenotypes.

The distribution of COPD severity in our cohort is comparable to what has been reported from other population-based studies, including individuals with predominantly mild- to moderate COPD [20, 32, 33]. COPD was based on post-bronchodilator spirometry, and both internal and external validity are considered good. In a population-based study, the results will not be affected by the under-diagnosis of COPD, allowing a discussion of generalizability to COPD in the general population.

Limitations of this study that merit further discussion are firstly that the analysis of MMP-9 and TIMP-1 includes both total and pro-enzyme levels. Secondly, serum MMP-9 levels may not reflect overall MMP-9 airway activity, since enzyme levels do not directly reflect enzyme activity. Further, in the present study, all samples have been collected during field studies and stored at − 20 °C. Thus, a decrease in MMP-9 levels across time, cannot be excluded [4]. The possible effects of storage are expected to affect all samples in an equivalent manner, as they were all collected within the same year, but the geometric decrease in measurable enzymes should be the same for all samples. The measured absolute values may be influenced by storage, but not likely the results, regarding the observed correlations. Furthermore, prognosis was assessed as all-cause mortality and the association between these biomarkers and specific cause of death would be of interest. However, unfortunately, data on cause-specific mortality were not available when this manuscript was completed.

## Conclusions

In this population-based study, we could not demonstrate the expected association between the measured biomarkers and lung function decline among subjects with COPD. This result may, after analyses of non-participation, be explained by a healthy survivor effect. Increased MMP-9 serum levels and MMP-9/TIMP-1 ratio, indicating increased proteolytic imbalance, were associated with mortality in both subjects with and without COPD. Among subjects with COPD, both these biomarkers were associated with an increased risk for death also when adjusted for common confounders. We propose that the prognostic value of systemic markers of increased proteolytic imbalance can be translated to a population level by this study, which includes predominantly mild and moderate COPD. Future longitudinal studies are important for the further understanding of MMP-9 and TIMP-1 in relation to the pathogenesis and prognosis of different COPD phenotypes.

## Additional file

Additional file 1: **Table S1.** Serum biomarkers in 2005, comparing subjects participating respectively alive but not participating in examination 2010. **Table S2.** Univariate Cox regression analyses of risk for death expressed as HR (95% CI), analyses stratified for non–COPD and COPD. **Table S3.** Risk for death expressed as HR (95% CI), analyses stratified for non-COPD and COPD in a Cox regression model adjusting for sex, age, smoking-status, heart disease and FEV_1_%. **Table S4.** Risk for death expressed as HR (95% CI), analyses for COPD in a Cox regression model adjusting for sex, age, smoking-status, heart disease and productive cough. **Table S5.** Risk for death expressed as HR (95% CI), analyses stratified for non-COPD and COPD in a Cox regression model adjusting for sex, age, pack-years and heart disease. (DOCX 23 kb)

## Abbreviation

AATD: Alpha-1 Antitrypsin Deficiency
COPD: Chronic Obstructive Pulmonary Disease
ELISA: Enzyme-Linked Immunosorbent Assay
FEV_1_: Forced Expiratory Volume in one second
FVC: Forced Vital Capacity
GOLD: Global Initiative for Obstructive Lung Disease
HR: Hazard Ratio
IQR: Interquartile Range
MMP-9: Matrix Metalloproteinase-9
OLIN: Obstructive Lung disease In Northern Sweden
SVC: Slow Vital Capacity
TIMP-1: Tissue Inhibitor of Metalloproteinases-1
VC: Vital Capacity
