# Supplementary material for: Proteolytic biomarkers are related to prognosis in COPD- report from a population-based cohort
Source: Respir Res. 2018 Apr 12;19:64. doi: 10.1186/s12931-018-0772-5 (PMC5897990; doi:10.1186/s12931-018-0772-5)
Supplement: Supplementary file 1 — Table S1. Serum biomarkers in 2005, comparing subjects participating respectively alive but not participating in examination 2010. Table S2. Univariate Cox regression analyses of risk for death expressed as HR (95% CI), analyses stratified for non–COPD and COPD. Table S3. Risk for death expressed as HR (95% CI), analyses stratified for non-COPD and COPD in a Cox regression model adjusting for sex, age, smoking-status, heart disease and FEV1%. Table S4. Risk for death expressed as HR (95% CI), analyses for COPD in a Cox regression model adjusting for sex, age, smoking-status, heart disease and productive cough. Table S5. Risk for death expressed as HR (95% CI), analyses stratified for non-COPD and COPD in a Cox regression model adjusting for sex, age, pack-years and heart disease. (DOCX 23 kb) [file 12931_2018_772_MOESM1_ESM.docx]

**e-Table 1. Serum biomarkers in 2005, comparing subjects participating respectively alive but not participating in examination 2010**

|  | **non-COPD** | |  | **COPD** | |  |  |  |
| --- | --- | --- | --- | --- | --- | --- | --- | --- |
|  | **Non-participants n=262** | **Participants n=602** | **P^1^** | **Non-participants n=165** | **Participants n=350** | **P^1^** | **P** ^2^ | **P** ^3^ |
| MMP-9 (ng/ml), median (IQR) | 528 (442-612) | 485 (216-601) | **<0.001** | 577 (479-657) | 459 (133-623) | **<0.001** | **0.003** | 0.525 |
| TIMP-1 (ng/ml), median (IQR) | 287 (236-336) | 344 (210-602) | **<0.001** | 292 (241-344) | 319 (214-571) | **0.004** | 0.753 | 0.218 |
| MMP-9/TIMP-1-ratio, median (IQR) | 1.79 (1.39-2.35) | 1.05 (0.65-1.86) | **<0.001** | 1.88 (1.50-2.54) | 1.02 (0.56-2.09) | **<0.001** | 0.238 | 0.504 |
| Deceased individuals excluded from the analyses. Significant p-values in bold.  P**^1^** comparing non-participants and participants in non-COPD respectively COPD  P^2^ comparing non-COPD and COPD among non-participants.  P^3^ comparing non-COPD and COPD among participants. | | | | | | | | |

**e-Table 2. Univariate Cox regression analyses of risk for death expressed as HR (95%CI), analyses stratified for non–COPD and COPD.**

|  | **Non–COPD** | **COPD** |
| --- | --- | --- |
|  | **Hazard–ratio (95% CI)** | **Hazard–ratio (95% CI)** |
| Sex (female^1^) | 1.868 (1.197–2.913) | 1.488 (0.937–2.362) |
| Age^2^ | **1.102** (1.077–1.128) | **1.104** (1.076–1.132) |
| Pack years^2^ | 1.028 (1.013–1.042) | 1.007 (0.994–1.019) |
| Ex–smoker (non-smoker^1^) | **2.586** (1.594–4.195) | 1.198 (0.678–2.115) |
| Current smoker (non-smoker^1^) | 1.915 (0.958–3.830) | 1.184 (0.655–2.142) |
| Productive cough, prc (no prc^1^) | 1.348 (0.843–2.155) | **1.696** (1.097–2.623) |
| Heart disease (no heart disease^1^) | **3.231** (2.141–4.877) | **2.318** (1.505–3.572) |
| FEV_1_ % predicted 2005^2^ | 0.216 (0.044–1.058) | **0.035** (0.010–0.119) |
| MMP–9^2^ | **1.002** (1.001–1.003) | **1.003** (1.002–1.004) |
| TIMP–1^2^ | 0.999 (0.998–1.000) | 0.999 (0.998–1.000) |
| MMP–9/TIMP–1 ratio^2^ | **1.183** (1.070–1.307) | **1.343** (1.133–1.591) |
| 1: Binary variable. 2: Continuous variable. Significant values in bold. | | |

**e-Table 3. Risk for death expressed as HR (95%CI), analyses stratified for non-COPD and COPD in a Cox regression model adjusting for sex, age, smoking-status, heart disease and FEV_1_%**

|  | **Non-COPD** | **COPD** |
| --- | --- | --- |
|  | **n=948** | **n=594** |
|  | **HR (95% CI)** | **HR (95% CI)** |
| Female | 1 (–) | 1 (–) |
| Male | **1.687** (1.06–2.686) | 1.486 (0.898–2.459) |
| Age^1^ | **1.106** (1.079–1.135) | **1.113** (1.082–1.144) |
| Non-smoker | 1 (–) | 1 (–) |
| Ex-smoker | **2.16** (1.305–3.574) | 1.068 (0.576–1.981) |
| Current smoker | **4.897** (2.341–10.245) | **2.201** (1.164–4.16) |
|  |  |  |
| No heart disease | 1 (–) | 1 (–) |
| Heart disease | **2.337** (1.492–3.659) | 1.19 (0.737–1.922) |
| FEV_1_% | 0.486 (0.11–2.143) | **0.064** (0.017–0.232) |
| MMP-9 ^2^ | 1.001 (1–1.003) | **1.003** (1.001–1.004) |
| TIMP-1 ^2^ | **0.997** (0.996–0.999) | 0.998 (0.997–1) |
| MMP-9/TIMP-1-ratio ^2^ | **1.105** (1.018–1.2) | **1.291** (1.072–1.556) |
| 1: Continuous variable. 2: Added one by one to the multivariate model. Significant values in bold. | | |

### e-Table 4. Risk for death expressed as HR (95%CI), analyses for COPD in a Cox regression model adjusting for sex, age, smoking-status, heart disease and productive cough.

|  | **COPD** |
| --- | --- |
|  | **n=594** |
|  | **HR (95% CI)** |
| Female | 1 (–) |
| Male | 1.54 (0.933–2.539) |
| Age | **1.112**  (1.082–1.144) |
| Non-smoker | 1 (–) |
| Ex-smoker | 1.296 (0.709–2.372) |
| Current smoker | **2.433** (1.274–4.646) |
| No heart disease | 1 (–) |
| Heart disease | 1.361 (0.843–2.197) |
| Productive cough | 0.995 (0.625–1.584) |
|  |  |
| MMP-9^1.2^ | **1.003**  (1.002–1.004) |
| TIMP-1^1.2^ | 0.998 (0.997–1.000) |
| MMP-9/TIMP-1-ratio^1.2^ | **1.306**  (1.088–1.568) |
| 1: Continuous variable. 2: Added one by one to the multivariate model. Significant values in bold. | |

### e-Table 5. Risk for death expressed as HR (95%CI), analyses stratified for non-COPD and COPD in a Cox regression model adjusting for sex, age, pack-years and heart disease.

|  | **Non-COPD** | **COPD** |
| --- | --- | --- |
|  | **n=948** | **n=594** |
|  | **HR (95% CI)** | **HR** |
| Female | 1 (–) | 1 (–) |
| Male | **1.739 (**1.092–2.767) | 1.436 (0.891–2.317) |
| Age^1^ | **1.105** (1.077–1.132) | **1.104** (1.075–1.133) |
| Pack years^1^ | **1.030** (1.016–1.044) | 1.009 (0.997–1.021) |
| No heart disease | 1 (–) | 1 (–) |
| Heart disease | **2.279** (1.468–3.540) | 1.239 (0.765–2.008) |
|  |  |  |
| MMP-91.2 | 1.001 (1.000–1.002) | **1.003** (1.002–1.005) |
| TIMP-11.2 | **0.998** (0.996–0.999) | 0.998 (0.997–1.000) |
| MMP-9/TIMP-1-ratio1.2 | 1.082 (0.998–1.174) | **1.357** (1.137–1.620) |
| 1: Continuous variable. 2: Added one by one to the multivariate model. Significant values in bold. | | |
